# Improving Access to Emergency Contraception Pills through Strengthening Service Delivery and Demand Generation: A Systematic Review of Current Evidence in Low and Middle-Income Countries

**DOI:** 10.1371/journal.pone.0109315

**Published:** 2014-10-06

**Authors:** Angela Dawson, Nguyen-Toan Tran, Elizabeth Westley, Viviana Mangiaterra, Mario Festin

**Affiliations:** 1 World Health Organization Collaborating Centre for Nursing, Midwifery and Health Development, Faculty of Health, University of Technology, Sydney (UTS), Sydney, New South Wales, Australia; 2 School of Public Health and Community Medicine, University of New South Wales, Sydney, Australia; 3 International Consortium for Emergency Contraception, New York, New York, United States of America; 4 RMNCH and HSS Technical Advice & Partnerships Department, The Global Fund to Fight AIDS, Tuberculosis and Malaria, Vernier-Geneva, Switzerland; 5 Department of Reproductive Health and Research, World Health Organization, Geneva, Switzerland; NHS lothian and University of Edinburgh, United Kingdom

## Abstract

**Objectives:**

Emergency contraception pills (ECP) are among the 13 essential commodities in the framework for action established by the UN Commission on Life-Saving Commodities for Women and Children. Despite having been on the market for nearly 20 years, a number of barriers still limit women's access to ECP in low- and middle-income countries (LMIC) including limited consumer knowledge and poor availability. This paper reports the results of a review to synthesise the current evidence on service delivery strategies to improve access to ECP.

**Methods:**

A narrative synthesis methodology was used to examine peer reviewed research literature (2003 to 2013) from diverse methodological traditions to provide critical insights into strategies to improve access from a service delivery perspective. The studies were appraised using established scoring systems and the findings of included papers thematically analysed and patterns mapped across all findings using concept mapping.

**Findings:**

Ten papers were included in the review. Despite limited research of adequate quality, promising strategies to improve access were identified including: advance provision of ECP; task shifting and sharing; intersectoral collaboration for sexual assault; m-health for information provision; and scale up through national family planning programs.

**Conclusion:**

There are a number of gaps in the research concerning service delivery and ECP in LMIC. These include a lack of knowledge concerning private/commercial sector contributions to improving access, the needs of vulnerable groups of women, approaches to enhancing intersectoral collaboration, evidence for social marketing models and investment cases for ECP.

## Introduction

Universal access to sexual and reproductive health and rights is an essential component of a healthy society. There are 222 million women in the world who wish to prevent pregnancy but are not using effective, modern methods of contraception. This results in an estimated 86 million unplanned pregnancies, 33 million unplanned births [1] and 20 million unsafe abortions every year [2]. In addition there are nearly 15 million births to adolescent women aged 15–19 each year, over 90% are in low and middle income countries (LMIC) [3]. Complications from pregnancy and birth are the leading causes of death for young women [4] often linked to a lack of access to service, information and care [5].

Strengthening service delivery and demand generation are key strategies to achieving universal access to sexual and reproductive health (SRH), including access to Emergency Contraceptive Pills (ECP). ECP is included in the list of 13 essential commodities in the framework for action established by the UN Commission on Life-Saving Commodities for Women and Children (UNCoLS) [6]. ECP is a safe method of preventing pregnancy after sex has occurred and can be easily provided to and used by women (unlike the intra-uterine device or IUD, another emergency contraception method which requires specialist skills for insertion) [7]. Despite having been on the market for close to 20 years, a number of barriers still limit women's access to ECP [8] in low- and middle-income countries (LMIC), including limited consumer knowledge, social marketing efforts, and inclusion in national family planning programs [9].

Quality service delivery at primary health care level, in both public and non-state sectors, is dependent upon inputs into the health system including supportive policies, evidence-based guidelines, trained workforce, secure contraceptive procurement and supplies, and financing. Ensuring the availability and access to quality health services is one of the main functions of a health system [10]. However in order to address the complex multi-dimensional determinants of SRH and ensure that services are convenient for users and efficiently managed, SRH should be integrated within a system that offers services, care and information across the health sector [11] and beyond. Inter-sectoral action for SRH service provision can take place across the health, education, media and justice sectors [12] and can engage both government, NGO and commercial enterprises [13].

In order to improve the service delivery of ECP, leaders in LMICs require evidence to inform country efforts to strengthen quality assurance mechanisms, build the capacity of the pharmacy/drug-selling sectors to provide high-quality service and advice, co-ordinate and scale-up service provision, and generate demand through interventions such as social marketing. Improving service delivery requires building the knowledge base at country level. However, there is a paucity of such knowledge, nor is there a synthesis of quality evidence from a number of nations available to decision-makers in their efforts to increase access to ECP.

In order to provide evidence to contribute to strengthening ECP delivery channels and developing policy guidance and tools to expand access to ECP including among vulnerable groups, we undertook a narrative synthesis of current research. The aim of this review was to identify what service delivery opportunities and interventions in LMIC can facilitate increased access to ECP. The results of this review and related reviews into consumer and service provider experiences will provide evidence to assist the ECP Technical Reference Team that has been established to help carry forward the UNCoLS recommendations at the global and national levels [14] as well as other efforts to expand access to ECP in LMICs.

## Method

A narrative synthesis methodology was employed to analyse selected literature. This method was chosen due to the range of qualitative and quantitative methodologies of the studies identified for the review, which did not allow the pooling of results that are achieved in other systematic approaches to synthesising evidence such as statistical meta-analysis [15]. The narrative synthesis was carried out in six phases [16] that when closely followed have been found to ensure transparency and reproducibility of findings [17], [18]. These steps first involved identifying the focus of the review and mapping current literature in the field through a scoping activity. This allowed the review question to be clearly defined enabling the development of specific selection criteria for the literature search. To be included in the review studies were assessed to see whether they met the required criteria. The quality of the selected studies were then appraised according to established tools and then data extracted from the findings sections to identify detailed information relating to the interventions to increase access to ECP. Finally the findings from the extraction were brought together to reveal patterns that were grouped into various sub groups. This process is described in more detail below.

The scoping exercise identified databases and website where ECP literature could be retrieved and potentially useful keywords identified. Seven bibliographic databases (CINAHL, MEDLINE, PubMed, SCOPUS, ProQuest Health & Medical Complete, Web of Science, African Journals On Line), meta-Indexes (Popline, Eldis knowledge services, Reproductive Health Library); websites of relevant organizations (The Guttmacher Institute, The International Consortium on Emergency Contraception, Population Council); Google Scholar and the reference lists of key papers were searched to retrieve research literature.

A Population, Interventions, Comparators, Outcomes, Study design (PICOS) question was formed to guide this review as per guidelines [19]. A PICOS question is an approach to designing a research question in order to identify specific evidence to inform clinical or health service practice. The question has five components that describe the population or participants that are the focus of study, the interventions, the control or comparison, the outcomes and the study design. In this review a control or comparison was not relevant. The PICOS question for this review was: For women of reproductive age who wish to prevent pregnancy what health service interventions have led to an increase in access to ECP in LMICs? Access to ECP was defined as the factors that facilitate the ability of women to make a choice about the use of ECP to prevent pregnancy and understood according to four aspects of availability (sufficient quantity), affordability (financial), physical accessibility and socio-cultural acceptability of product and supply method [20]. This definition also draws on other frameworks related to access, including work from Frost and Reich [21], WHO's equitable access to essential medicines [22], and Ensor and Cooper's demand-side barriers [23]. The review objective was to identify what and how service delivery interventions and/or strategies had addressed these factors to facilitate access to and potential utilization of ECP. We anticipated that ECP service delivery interventions may have an impact on factors such as: consumer knowledge, attitudes and practice; provider knowledge, training outcomes, and practices; and uptake of ECP particularly among vulnerable women such as such as adolescents and sex workers. The definition of service delivery was guided by the WHO Health System Building Blocks [24].

Observational studies, quasi experimental and non-experimental descriptive studies were considered suitable for inclusion and a systematic search of the contemporaneous primary research literature published from 2003 to 2013 in English in LMIC was undertaken. Electronic databases and the internet were searched using Medical subject headings (MeSH) (America's National Library of Medicine's controlled vocabulary thesaurus). The following terms were employed: ‘Postcoital Contraception’ and ’Health Services Accessibility’ and ‘Healthcare Delivery’ and ‘Contraceptive Distribution’ and ‘Developing Countries’ and supplemented by the terms key words ‘emergency contraception,’ or ‘emergency contraceptive pills’ using the MESH terms, abstract and keyword options. This was modified as returns were poor. For example a search of ProQuest Health & Medical Complete (Postcoital Contraception) AND MeSH (’Health Services Accessibility) AND MeSH (Healthcare Delivery) AND MESH (Contraceptive Distribution) AND MeSH (Developing Countries) AND ab((emergency contraception OR emergency contraceptive pills))returned no results. Searches that returned results are outlined in Table 1 alongside those identified for appraisal.

**Table 1:** Record retrieved from databases and internet sites.

Source	Search terms	retrieved	Identified for appraisal
**Databases**			
PubMed	(Postcoital Contraception[MeSH Terms]) AND Health Services Accessibility[MeSH Terms]) AND Healthcare Delivery[MeSH Terms]) AND Contraceptive Distribution[MeSH Terms]) AND Developing Countries[MeSH Terms]) OR emergency contraception[Title/Abstract]) AND emergency contraceptive pills[Title/Abstract]	70	0
Medline	((Postcoital Contraception and Health Services Accessibility and Healthcare Delivery and Contraceptive Distribution and Developing Countries).sh. or emergency contraception.ab.) and emergency contraceptive pills.ab.	45	0
SCOPUS	ABS(emergency contraceptive pill)	233	0
ProQuest Health & Medical Complete	ab((emergency contraception OR emergency contraceptive pills))	396	1
CINHAL	AB emergency contraception	246	0
Web of Science	TOPIC**:** (emergency contraception pills)	439	
**Websites**			
Africa Journals On-Line	emergency contraception pill	23	1
Popline	Advanced search 2003–2013 English Emergency contraception research report	772	2
Eldis knowledge services	Emergency contraception	471	2
RHL Reproductive Health Library	Emergency contraception	10	0
The Guttmacher Institute	Emergency contraception	303	0
Population Council	Emergency contraception	39	9
The International Consortium on Emergency Contraception	Emergency contraception	53	8
**Hand searching**		4	2
Minus duplicates			9
Total		3104	16

**Figure 1:**
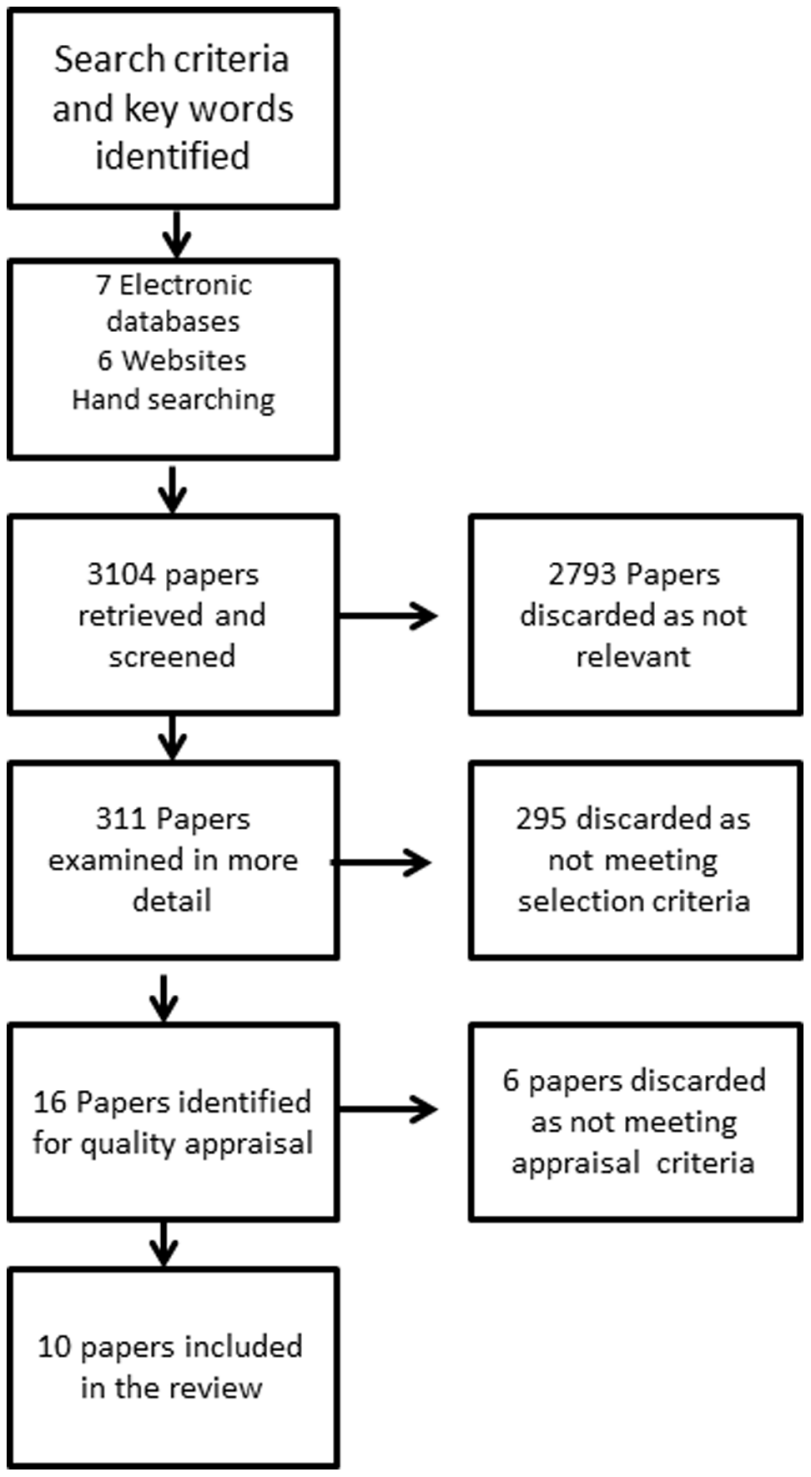
Overview of the literature review process.

Retrieved records were first screened for their focus as per the PICOS question by the first author and duplicates removed. Discursive papers (papers without research data), those older than 10 years or whose focus was outside of the aim were removed. Preferred Reporting Items for Systematic Reviews and Meta-Analyses (PRISMA) guidelines were used to report the review process [25] PRISMA is an evidence based minimum set of items for the transparent and complete reporting of systematic reviews and consists of a 27 item checklist and flow diagram of four phases. The flow chart is illustrated at Fig 1 and shows the numbers of items retrieved and then discarded. An inclusion/exclusion criterion was applied to 311 papers; of these 295 were discarded because the focus was not on a detailed description of the health service intervention, rather the study factor was consumer or provider views and experiences. Papers were carefully assessed for their relevance to the definition of access to health service as described above.

Sixteen papers were identified as relevant in discussion with all authors. These papers were appraised by all authors using the Critical Appraisal Skills Programme (CASP) tool, a checklist developed for assessing the quality of qualitative evidence for use in policy and practice [26]. Pluye et al.'s [27] scoring system was used to assess the quality of non-experimental studies and mixed methods. Research reports were classified as grey literature and were appraised using a checklist developed for this purpose [28].

Six items were discarded: reviews that did not include primary data [29]–[31]; project briefs with scant information on methods and primary data [32], [33]; and a report [34] that formed the basis for a peer reviewed paper that was included in the synthesis [35].

A narrative synthesis approach was conducted as per guidelines [16] allowing different data from diverse traditions to be examined to provide critical insights. The results sections of each of the 10 papers were imported into QSR NVivo 10 software, a qualitative research management tool. The characteristics of all studies were first explored including the context and method. Tables were used to assist in the data extraction process and the contents of the cells discussed among authors. We then undertook a textual narrative approach to the analysis of the study findings based on worked examples provided by Lucas et al. [36]. This involved a commentary approach to examine similarities and differences among the interventions outlined in the studies included in the review. A published review describing categories of strategies found to improve the coverage, access and quality of health services in developing countries [37], [38] helped to focus the textual analysis on the ECP service delivery interventions and their specific elements. The relationships within and between interventions described in the findings section of studies included in the review were explored and commentaries written by the first author. Concept maps were used to plot patterns and relationships and robustness assessed through critical reflection and discussion among the authors.

## Results

**Figure 2:**
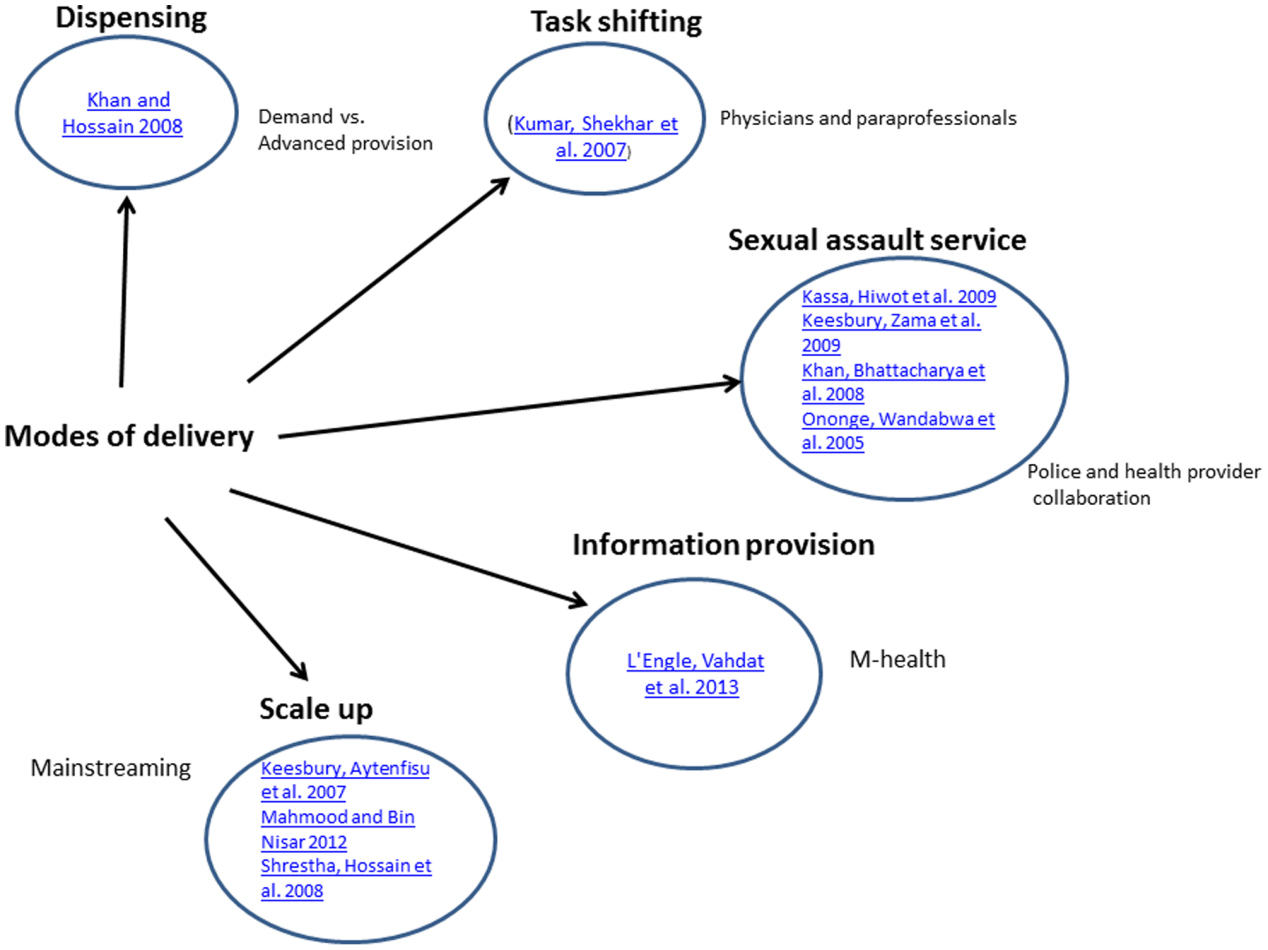
Approaches to ECP service delivery.

Ten documents were included in this study (see Table 2). Five documents outline studies in Africa (Uganda, Tanzania, Zambia and two from Ethiopia) and 5 from Asia (Bangladesh, Nepal, Pakistan, India and 1 involving data gathered from both India and Bangladesh). Mixed methods were employed in 8 studies while 2 studies involved descriptive quantitative surveys. The analysis identified different modes or approaches of ECP delivery. Figure 2 outlines the key focus of the interventions for the 10 studies included in the review. Several studies were grouped under one intervention mode and some studies included multiple interventions allowing comparisons to be made across those that shared similar characteristics.

**Table 2:** Summary of studies included in the Review.

Reference	Context	Method	Sample/participants	Aim	Findings
*ECP and sexual assault services*					
(Kassa, Hiwot et al. 2009)	Ethiopia Addis Ababa, hospitals	Mixed methods- Descriptive cross sectional survey of all health facilities in Addis Ababa to assess ECP provision after sexual assault. In-depth interview were conducted with key informants at police stations.	576 health facilities in Addis Ababa and 4 police stations	To examine the potential barriers to accessing ECP among sexual assault survivors	Five public hospitals and one model clinic (1.04% of all facilities) provide treatment to victims of sexual assault and provide ECP. No private hospital provides treatment. Low police knowledge of ECP and referral usually to model clinic. Lengthy processing times and cost to women make court action difficult.
Keesbury, Zama et al. 2009)	Zambia Ndola district	Mixed methods intervention study. Descriptive quantitative: service provision data from police stations, provider KAP survey. Qualitative: focus group discussions and key informant interviews.	210 police officers were interviewed from 15 police stations and posts, 3 health workers, 1 health official, 3 community members	Assess the feasibility of police provision of ECP	Police can safely and effectively provide ECP. Reporting of sexual violence cases increased by 48% in participating police stations from 2006 to 2007. The program was perceived by provincial management as successful, sustainable and cost-effective.
(Khan, Bhattacharya et al. 2008)	India (Delhi, Lucknow, Vadodara) Bangladesh (Dhaka, Chittagong, Sylhet Tangail)	Mixed methods Descriptive quantitative: Survey of providers and open questions	44 medical practitioners and nurses in public hospitals and 55 police in stations adjacent to medical facilities	To assess post-rape care services available at the first point of contact	No uniform service provision protocol to follow for managing rape survivors at health facilities.
(Ononge, Wandabwa et al. 2005)	Mulago Hospital, Kampala, Uganda	Quantitative descriptive study using survey and clinical records	Fifty eight sexually assaulted females were interviewed, examined, given appropriate treatment and followed up for 3 months	To determine the presentation and treatment offered to sexually assaulted females attending emergency gynaecological ward	The mean age was 9.5 with a range of 1–35 years. ECP offered to all women and girls. STIs included trichomonas vaginalis (1.7%) and syphilis (3.7%). All cases received counselling and prophylactic treatment for sexually transmitted infections
*Scaling up and mainstreaming ECP*					
(Keesbury, Aytenfisu et al. 2009)	Ethiopia: Addis Ababa, Oromiya, Amhara, SNNPR, and Tigray	Mixed methods intervention study. Descriptive quantitative: Facility data of clients who received ECP may 2006-Dec 2006 and survey of client KAP, survey of providers to assess their experiences with ECP before and after and qualitative interviews held.	33 facilities and 121 providers from the five regions: nurses (73%) midwives (14%); 3 doctors, health centre clients interviewed, 3999 cases of ECP use	To assess feasibility of expanded access to ECP in the public system.	At start of the project 20% clients had heard of ECP and 0.03% had used ECP. Utilization of ECP steadily increased throughout the project period. Providers were significantly more likely to report a preference to deliver ECP services to adolescent girls than to adolescent boys. Support for expanded access at emergency rooms and police stations, schools and CHWs.
(Mahmood and Bin Nisar 2012)	Pakistan DG. Khan, Gawadar, Mansehra, Thatta	Intervention study using quantitative descriptive survey	193 Lady Health Workers (LHWs), 2,093 married women of reproductive age	To understand and measure retention of knowledge, attitudes and practices of the trained HWs and married women of reproductive age	LHWs (94%) stated the correct answer regarding the use of ECP, <75% stated that they provided the ECP to all the women who required ECP, 47% reported that they did not receive ECP doses from their department on a regular basis, 35% kept a record of the ECP clients, 11% referred their clients to the nearest health facilities for ECP, 87% contacted ECP clients after giving them doses, 81% of women had heard about the ECP, 29% reported ever using ECP.
(Shrestha, Hossain et al. 2008)	Nepal: Kathmandu, Bhaktapur and Lalitpur	Mixed methods intervention study. Surveys of providers, facility records, interviews with women, observations of client-provider interactions	545 service providers, 60 ECP users, 47 client-provider interactions	To describe the introduction of ECP into the national family planning program	Use of ECP was lower than expected during the nine-month intervention period. Limited availability only at health centres. Quality of ECP services was reported to be satisfactory among clients, leaflets shared widely
*Task shifting for ECP*					
(Kumar, Shekhar et al. 2007)	India: Meerut (Uttar Pradesh), Jaipur (Rajasthan), and Thane (Maharashtra)	Mixed method intervention study. 2 intervention sites and a control. Service data of women receiving ECP, survey of providers, survey of women and FGDs.	6 CH Centres per site (2 intervention a, 2 intervention b, 2 control). 146 physicians, 593 paraprofessionals, 409 women in intervention 1, 766 women in 2, two follow-up visits with 316 women	To compare two different ECP delivery models: physicians compared with physicians and paraprofessionals.	Increase in provider and client ECP knowledge in intervention site at 9 months. At 6 months women who received ECP reported level of counselling better among paraprofessionals than physicians.
*Models of dispensing ECP*					
(Khan and Hossain 2008)	Bangladesh Tangail and Mymensingh, Dhaka division	Mixed method intervention study. Descriptive qualitative: FGDs, KIIs and in depth interviews Descriptive quantitative: Survey of providers, client survey	36 women 17 men, 54 married women interviewed, 290 providers, 3,900 female clients (1, 300 each from the prophylactic, on-demand and control areas)	To test the relative effectiveness of two alternative service delivery models for providing ECP: on demand vs in advance.	Increase in provider and client knowledge of ECP, study demonstrated high acceptability of ECP. The model providing ECP as a prophylactic was far more successful in meeting the needs of the clients for ECP than the model which provided ECP on-demand after unprotected intercourse occurred. If used correctly, the success rate in avoiding unwanted pregnancy was 99%.
*ECP Information Provision to the Community*					
(L'Engle, Vahdat et al. 2013)	Tanzania	Mixed methods. Survey and open questions via text message.	2870 unique m4RH users during the pilot period	To evaluate the feasibility, reach and potential behavioural impact of providing automated family planning information via mobile phones to the general public	Sixty percent were 29 or younger years in age. ECP was the second most popular method queried.

### ECP and sexual assault services

ECP service delivery to survivors of sexual assault was examined in four studies [39]–[42]. Ononge et al. [42] documents the services and commodities sexual assault survivors received at Mulago National Referral Hospital Uganda. These included the provision of combined oral contraceptive pills as emergency contraception, vaginal swabs, HIV and sexually transmitted infection (STI) testing and counselling, and antibiotics and surgery to repair genital trauma. HIV prophylaxis was not offered because it was not available. A descriptive study in Ethiopia found that 6 (5 public hospitals and 1 clinic) of the 576 health facilities surveyed in Addis Ababa provided services to survivors of sexual assault [39]. Almost all survivors of sexual assault presented to those health facilities with a police letter certificating the amount and type of injury sustained. Public hospitals provided a dedicated ECP (i.e.‘Postinor 2’ containing two 0.75 mg levonorgestrel), on site and services free of charge 24 hours a day, 7 days a week. One clinic was open 8-5 Monday to Friday and on Saturday mornings and provided prescriptions that enabled women to buy ECP at the clinic pharmacy for five Birr (0.5 USD), or free of charge if the women could not afford it. In the Zambian study in this review it was noted that no fees were charged to rape survivors for any associated procedure, test, or investigation at health facilities [40]. However issues were identified concerning the psychosocial care and services for child survivors and at health facilities, especially in regard to documentation and forensic evidence collection due to staff turnover and rotations [40]. Another descriptive study in the review [41] investigated the provision of sexual assault services in India and Bangladesh and found a lack of uniform service provision and protocols for managing rape survivors at public hospitals. STI management was not available in most health facilities, and where services were available regimens were not standardized. Referrals to voluntary HIV counselling and testing centres were uncommon and most health professionals were not aware of HIV Post-Exposure Prophylaxis.

The study by Khan et al. also examined ECP service provision at police stations in India and Bangladesh and also found a lack of consistent protocols and low police knowledge of ECP [41]. All police personnel interviewed in India and 48% in Bangladesh admitted to never discussing the possibility of unwanted pregnancies and nearly all police respondents were unfamiliar with ECP. None of the police stations had provisions for stocking ECP.

An intervention study involving 20 purposefully selected police stations in urban and peri-urban study sites in Zambia demonstrated promising results of stocking ECP at police stations and training police officers to deliver ECP to eligible survivors of sexual violence and high rates of referral of women to health facilities [40]. Over a three year period police officers in all 20 stations provided a total of 357 doses of ECP to survivors of sexual violence. No adverse events or complaints were reported in any of these cases. Some challenges to provision were noted including high turnover rates of trained police officers, poor handover training and costs incurred by both police and women to travel to health centres. In addition, stock outs at health facilities near police stations lead to referral by health workers to the police stations thereby reducing stock dedicated to survivors of sexual assault.

### Scaling up and mainstreaming ECP

Scaling up and mainstreaming ECP in national programs was the focus of three studies [35], [43], [44] (see Table 3). The findings of these studies show that scaling up and mainstreaming ECP within country national programs is feasible in Ethiopia, Nepal and Pakistan, including the involvement of community workers and paraprofessionals as found in the Indian study of task sharing, which is also described in this review [45]. Keesbury et al. [35] and Shrestha et al. [44] outline comprehensive scale up and mainstreaming initiatives that go beyond health provider training to include advocacy efforts, social marketing (Ethiopia only) and community education endeavours, as well as commodity provision. Data collected in both studies included health facility records of clients who were dispensed/prescribed ECP, providing insight into the nature of the complex intervention. The Ethiopian intervention study [35] involved the implementation of three strategies in 33 public hospitals and health centres as well as NGO facilities in five main regions that served as a pilot to inform plans to scale up ECP in public health facilities across the country. Key findings included a steady increase in the use of ECP in the study period, however provider preferences for supply to adolescent girls not boys were noted. The Nepalese pilot study [44] was concentrated on the delivery of 4 strategies in 3 districts in the most populated region in the country and involved all 135 primary health facilities excluding specialised hospitals. Uptake of ECP was found to be lower than expected which was attributed to availability at health centres only and the mountainous terrain. This study reports consultation and advocacy activities with stakeholders in key health service decision-making roles at national and municipal level beyond the development of training curricula. The study from Pakistan [43] involved an advocacy activity and the introduction of ECP through training community health workers. Although baseline data are not reported, three quarters of lady health workers reported providing ECP but issues were noted with procuring them for women due to limited stocks of ECP at health centres and individual provider record keeping. In these three studies ECP was made available free of charge during the period of the trial. Keesbury et al. [35] found that clients and providers felt an acceptable price for the sale of ECP sale in private pharmacies was 1 Ethiopian Birr (0.05 USD). In addition Keesbury et al. [35] describe the introduction, registration and importation of a dedicated ECP in Ethiopia as part of the intervention to ensure a continued commodity supply.

**Table 3:** Summary of scaling-up and mainstreaming interventions in studies in the review.

	Key strategies and aim
(Keesbury, Aytenfisu et al. 2009) Ethiopia	*Improve provider competency:* Doctors and nurses were trained, grants given to students to carry out small research projects on ECP and mentored.
	*Increase public demand:* Branding of packets with graphic and inclusion of condom, mass media campaign (TV, radio, newspaper and posters).
	*Ensure commodity security:* Postinor 2 approved for use and procured.
(Shrestha, Hossain et al. 2008) Nepal	*Advocacy:* Orientation for government officials and stakeholders.
	*Improve provider competency:* Training of trainers and of service providers; An orientation for Female Community Health Volunteers.
	*Increase public demand:* Implementation of educational activities; IEC materials developed and distributed.
	*Provision of ECP:* Postinor *2* introduced into normal contraceptive delivery system marked “Only For Free Distribution”.
	*Improve monitoring and evaluation:* ECP included in service providers reports and pooled at district and central level.
(Mahmood and Bin Nisar 2012) Pakistan	*Advocacy:* Study tour to inform government officials and stakeholders.
	*Improve provider competency:* training of trainers and Lady health workers.

All three studies included provider training activities that were found to have improved knowledge. In Ethiopia [35] these involved two rounds of technical training for 190 doctors, midwives and nurses drawing on Population Council materials developed in Bangladesh and modified according to the findings of a baseline study of provider knowledge and input from key stakeholders. The final curricula were endorsed by the Ministry of Health. Research skills of doctors, nurses and community health students were also fostered through a funding completion resulting in the completion of 10 studies that contributed increased knowledge of issues relating to access and service delivery. In Nepal [44] the training involved a cascade model where trainers were trained to deliver a one day training courses to 545 providers at health facilities. This curriculum was also derived from manual developed by the Population Council in Bangladesh and India. In addition to training materials a job aides in the form of an ECP information poster and brochure was developed for providers in clinics. Unlike the Ethiopia intervention Nepalese female community health volunteers also received an orientation to ECP in order to improve the referral of women and deliver education at the household level. However this activity was not evaluated. The Population Council study in Pakistan [43] also employed a cascade training model based on their previous experience in Bangladesh but focused only on Lady Health workers who provide services at household level. However unlike the Nepalese female health workers, Lady Health Workers distributed ECP and receive a salary from the health department.

### Task shifting and ECP

Task shifting and sharing was the key focus of one intervention study from India that investigated the difference between ECP service provision by paraprofessionals (lady health workers and auxiliary nurse midwives) and physicians [45]. Physicians were given ECP training and provided with health education material in a randomly selected intervention area. In another area, physicians and paraprofessionals received the same ECP training and materials. The matched control areas received no intervention. Service statistics showed that the number of ECP services provided to women reporting unprotected sex was higher in the area where physicians and paraprofessionals had been trained than in the area where just physicians where trained, or where no training had taken place. Women initiated the first dose of ECP sooner in areas where both cadres had been trained and dispensed ECP (with a mean of 37.3 hours, SD 19.2). The study therefore concluded that training both paraprofessionals and physicians can increase access to ECP in India. Task sharing with community health workers for ECP provision, education and referral is a feature of other studies in the reviews [43], [44] well as with other professionals such as the police [40] however the performance of these professionals was not compared with others.

### Models of dispensing ECP

One intervention study in the review is concerned with examining the effectiveness of providing ECP to women on demand compared with in advance in Bangladesh [46]. In the on-demand study group, all family planning clients received counselling and a brochure on ECP and were invited to attend the clinic for ECP supplies if needed. In the advanced-provision study group, the women received the same information but were provided with two packets of ECP to use in case of unprotected sex (during an emergency). A control group received no intervention. In the two study groups service providers (public, NGO sector and lay health workers) were trained. The findings show improvements in knowledge concerning ECP among both consumers and providers. Acceptability of ECP was reported to be high and mediated by socio-cultural variables and use found to be higher in the advanced-provision area than in the on-demand area. However, only about half of the ECP users used it correctly within the designated time period.

### ECP Information Provision to the Community

Print and electronic media were used to communicate messages about ECP in studies included in the review. In Nepal an ECP brochure was developed for clients that were found to have been widely shared in the community [44]. In Ethiopia the mass media was used as well as posters [35] to convey information about ECP as a safe and effective means of preventing pregnancy. These included television infomercials and spots on women's talk shows as well as infomercials on radio and in newspapers. Although detailed information about the effects of these mass media activities is not available in the paper the authors conclude that the increase in client's knowledge of ECP within the past year corresponded to the project's media outreach phase. Gaps in the provision of educational material for survivors of sexual assault was noted in Khan et al. study [41] where it was noted that police stations in India and Bangladesh did not have any informational handouts, such as leaflets or brochures, about ECP or reproductive health for women. L'Engle et al. [47] descriptive study is the only piece of research that was retrieved in this review that examined the feasibility of disseminating information using mobile phones to improve the provision of contraceptive information including ECP. In a Tanzanian text messaging campaign to provide information about different contraceptive methods via SMS, ECP was the second most queried method of contraception with 21% of queries. The majority of participants requesting information were under 29 years.

## Discussion

This review has identified limited knowledge concerning service delivery interventions in LMIC that can facilitate increased access to ECP. Data showing the effects of interventions on reproductive health outcomes [48], is not available for any of the initiatives examined and therefore the long term impacts of the interventions are unknown. In addition, the influence of the non-state sector is not noted in these studies included in this review. No papers were identified that addressed the role of the commercial or social marketing sectors in increasing access to ECP, despite the increasing importance of these organizations. In addition the quality of papers was found to be poor with a large number of documents excluded due to absent or low quality methods and primary data. The review also identified a lack of insight concerning ECP service delivery in relation to other aspects of the health system such as data gathering, policy, commodities and financing.

The findings of four descriptive studies included in the review indicate that considerable gaps exist in terms of service provision to survivors of sexual assault and rape. Despite one promising study more research is needed to determine how police and health workers can best collaborate to ensure survivors of sexual violence are treated with empathy and dignity and scale up the efficient quality care they require. Task shifting and sharing with cadres other than doctors was found to be a useful strategy to improve ECP access in four of the included studies, forming the focus of one study intervention [45] and appearing in the findings of three other studies [41], [43], [44]. However, provision of ECP in pharmacies by pharmacists and pharmacy clerks was not well-documented in the literature, despite the prevalence of this mode of service provision. Advanced provision of ECP was found to be more successful in meeting the needs of women than on-demand supply after unprotected sex as measured by reported use of ECP. However, as these are self-reported data and only one study could be located these results are inconclusive. The findings of this study differ from those in high income countries that show negligible effects of advanced supply on ECP use after unprotected sex, sexual activity, or regular contraceptive use [49]–[51].

More research is needed to better understand how mobile phone technologies and other forms of electronic communication can be used to disseminate ECP information and respond to consumers' questions and how this can be integrated with other social marketing and education interventions.

There appears to be a dearth of comprehensive evidence of scale up and mainstreaming of ECP as only three studies were identified, none of which included evidence about all sectors that provide ECP. Evidence of workforce interventions is scarce particularly in terms of the types of workforce support and performance management initiatives that may be required across both the public and private sectors. This review highlighted that there is a paucity of evidence concerning what ECP data should be collected and best practice for commodity procurement and disbursement, financing for ECP, and ECP policy. As a result, there is little guidance for LMIC about how to prioritize the limited resources available for improving access to ECP. Little research learning has been documented and shared. This underlines the need to invest in strategic high quality research efforts to better understand what service delivery strategies work in specific contexts. There is also a need to identify lessons from successful (and unsuccessful) endeavours that can be applied by countries undertaking similar policy objectives as they relate to improving access to ECP.

Despite the commercial sector providing a significant portion of SRH health services in LMIC [52], and their particularly important role in the distribution of ECP, this review has found very little research in this area that provides evidence-based guidance for decision makers to best engage with the private sector, either commercial or social marketing, to improve access to ECP. Experiences such as the development of accredited drug dispensing outlets in Tanzania [53], and the expansion of private health provider networks and social franchises [54], [55] may be helpful to countries looking for innovative ideas to increase access to ECP. Lessons learned using diverse models to deliver other contraceptives may also provide insights for countries wishing to trial new approaches. Community-based distribution [56], [57] and community pharmacy supply [58] could provide timely access to ECP alongside other contraceptives. In terms of expanding and scaling up ECP, lessons can also be learned from other contraceptive research and applied to ECP studies in order to identify enabling factors and barriers to dissemination, diffusion, scale up, and/or sustainability [59]. This includes useful lessons from Latin America, Asia and Africa [29], [30], [60] illustrating the importance of regular evaluation and dissemination of findings to identify priority areas for improving contraceptive delivery, as well as the effect of health sector reforms on ECP promotion. In addition insights from supportive health service delivery efforts such as the Community-Based Health Planning and Service model adopted in Ghana to expand access to primary health care [61] could also serve to increase access to ECP.

### Limitations

This study may have been limited by an incomplete retrieval of research studies; however, efforts were made to search not only bibliographic databases but also meta-Indexes and the websites of international organizations. As a result more documents identified but despite meeting the appraisal criteria some of these may have been at a lower quality, as not all studies were published in peer reviewed journals. The application of a narrative synthesis to the results of the studies included in this review may have led to a loss of detail particularly of contextual factors that are important to the outcomes of the various interventions. However in this review textual descriptions of the studies provided a narrative that was maintained across all studies. The graphical elements included in the analysis were useful for identifying service delivery patterns and the tabulation of findings enabled structured comparisons across the interventions identified. A final limitation may have been our cut-off point of 10 years prior as more attention was paid to research on ECP introduction shortly after the dedicated product was made available, in the mid-late 1990s.

### Conclusion and recommendations for further research

ECP have been acknowledged as an essential commodity to prevent unwanted pregnancy and save women's lives; however women in LMIC face many barriers to accessing this contraceptive method. This review has identified a number of promising strategies to improving access including: advance provision of ECP; task shifting and sharing; intersectoral collaboration for sexual assault; m-health (the use of mobile devices such as mobile phones) for information provision and engagement of potential users; scale up through national family planning programs. However, the lack of high quality evidence resulting in few studies from which to glean comprehensive insights has led to the identification of a number of knowledge gaps. These gaps present a range of questions that should be the focus of research into how to best improve service delivery efforts for ECP. Table 4 presents these recommendations for further research that will provide decision makers in LMIC with much needed evidence to best design, implement and evaluate programs to increase access to ECP.

**Table 4:** Links between key areas of service delivery, review findings, gaps in knowledge and recommended focus for research.

Key areas of service delivery focus	Promising strategies based on findings	Gaps in knowledge	Areas for further research
Dispensing ECP	Advance provision of ECP.	Needs of vulnerable groups.	How can ECP be made available in public –private partnerships health sector settings?
		Impact of advance provision of ECP on contraceptive use.	How can ECP programs be tailored to meet the needs of the most vulnerable women?
		Costs to consumer.	What is the role of different sectors of the market? For example public sector/subsidized or free, social marketing/subsidized, commercial sector/not subsidized.
		Role of the private sector.	How can community networks be harnessed for ECP?
		Community based efforts.	What are the most effective user fee exemption fees and waivers for ECP?
Task shifting/task sharing	ECP delivery by CHWs, paraprofessionals and police.	Provision of ECP information, commodities and counselling in collaboration with other health professionals, social workers, teachers, youth workers etc.	How can effective Intersectoral collaboration be achieved across health education, media and justice sectors?
		Workforce performance measure.	How can task sharing and collaborative performance be assessed and incentivized?
Sexual assault service	Police training to dispense ECP and refer to health facilities, provision of sexual assault kits and at police stations and monitoring of activities.	Sustainability of interventions. Best practice protocols and standards across facilities.	What clinical practice guidelines and protocols need to be developed to ensure sexual assault services, information and care are provided at health facilities and how should this be implemented and monitored?
		Role of other agencies.	How women's shelters and other relevant agencies should be involved in the provision of ECP?
Information provision	Branding of ECP packets and social marketing through media campaigns. Engagement of decision makers. Mobile phones to deliver ECP information and advice.	Effects of information strategies over time to particular populations.	What social marketing initiatives best increase consumer demand and increase ECP access?
Scale up	Including ECP as part of national FP programs.	Costing models and investment cases for ECP, indicators for information gathering and coordination with other health programming	How can existing programs be used as opportunities to scale up ECP?
	Increase M&E efforts.		What approaches are most cost effective?
	Provider training.		How should ECP components of programs be monitored and evaluated and what impact data should be collected?
			How can ECP delivery be effectively integrated with STI/HIV/MNCH programs?

## Supporting Information

Checklist S1 **PRISMA checklist.**
(DOC)
